# Filial responsibilities and psychological wellbeing among Chinese adolescents in poor single-mother families: does parental warmth matter?

**DOI:** 10.3389/fpsyg.2024.1341428

**Published:** 2024-05-01

**Authors:** Janet T. Y. Leung, Daniel T. L. Shek

**Affiliations:** Department of Applied Social Sciences, The Hong Kong Polytechnic University, Kowloon, Hong Kong SAR, China

**Keywords:** filial responsibilities, adolescent psychological wellbeing, single-mother families, parental warmth, poverty

## Abstract

**Introduction:**

Adolescent children raised in single-mother households, particularly those living in poverty, often need to assume more instrumental and emotional familial responsibilities to cope with family challenges.

**Method:**

This study examined the relationships between these filial responsibilities and adolescent psychological wellbeing, as well as the moderating effect of maternal warmth on these relationships via survey. The sample comprised 325 Chinese adolescent children (43.3% girls; M_age_ = 13.5) from economically disadvantaged single-mother families in Hong Kong.

**Results:**

The results indicated that adolescents’ instrumental filial responsibilities were positively associated with their life satisfaction. Emotional filial responsibilities, on the other hand, were positively linked to life satisfaction and negatively associated with depression. Furthermore, maternal warmth was found to moderate the relationship between emotional filial responsibilities and life satisfaction. Adolescents who perceived higher levels of maternal warmth and performed more emotional filial responsibilities reported greater life satisfaction than those who performed fewer such responsibilities. Furthermore, the moderating effect of maternal warmth on the relationship between instrumental filial responsibilities and life satisfaction varied between boys and girls. Additionally, the age of the adolescent moderated the effect of maternal warmth on the relationship between emotional filial responsibilities and adolescent anxiety.

**Discussion:**

These findings suggest that filial responsibilities do not necessarily impede adolescent wellbeing. Instead, maternal warmth appears to be a crucial family factor that influences the nature of the relationship between filial responsibilities and adolescent wellbeing. These insights are valuable for family scholars and practitioners, informing the design of supportive services to enhance the psychological wellbeing of Chinese adolescents from economically disadvantaged single-mother families.

## 1 Introduction

Single motherhood has been found to have detrimental effects on family functioning and adolescent psychological wellbeing (Amato, 2005; Leung and Shek, 2016). Single mothers and their children often experience negative emotions such as resentment and depression, stemming from fractured marital and father-child relationships (Rice, 2001; Anderson, 2003). The challenges of recovering from such loss and maintaining family functioning in the future become the “personal and social baggage” of single-mother families (Anderson, 2003, p. 122). Moreover, research indicates that single-mother families are more susceptible to economic hardship. Single mothers encounter greater difficulties in securing employment due to their childcare responsibilities (Millar and Ridge, 2009). Even when they do find employment, their wages are typically lower than those of single fathers (Leung and Shek, 2016; Zagel and Hübgen, 2018). It is clear that the maternal distress, inadequate income, and poor parenting associated with single motherhood have detrimental effects on the wellbeing of their children (Jones et al., 2007).

The shift in family structure necessitates that adolescent children raised in economically disadvantaged single-mother families assume greater filial responsibilities (Nixon et al., 2012; Kuperminc et al., 2013). Filial responsibility is defined as the children’s efforts to support and assist their family (Fuligni and Zhang, 2004). There are two conceptual categories of filial responsibilities: instrumental and emotional. Instrumental filial responsibility refers to children’s duty to provide practical assistance to their parents, such as performing household chores and caring for younger siblings. Emotional filial responsibility involves children’s efforts to provide emotional comfort to their parents and siblings (Jurkovic et al., 2001; Kuperminc et al., 2013). Adolescents growing up in economically disadvantaged single-mother families are often required to share their mother’s burdens and concerns by assuming greater tangible and emotional responsibilities at home (Jurkovic et al., 2001; Burton, 2007). Furthermore, economic hardship often necessitates that they fulfill household duties and act as “surrogate parents” to their younger siblings (Sy and Romero, 2008; East, 2010). Unlike their peers, who can devote more time to developing their talents and socializing with friends, these adolescents must spend more time at home, assuming caregiving roles (Sy and Romero, 2008).

Existing literature, including parentification theory (Minuchin et al., 1967) and role identity theory (Fuligni and Flook, 2005), provides explanations for the relationship between filial responsibilities and psychological wellbeing among adolescents. Parentification theory posits that filial responsibilities can impede adolescent wellbeing as they are required to assume developmentally inappropriate parental roles within their family, which can be stressful (Chase, 1999). Conversely, social identity theory (Hogg, 2003) views family responsibilities as positive processes for adolescent children, as their contributions to their family can help them develop their family identity (Fuligni and Zhang, 2004). Therefore, this study aimed to examine the relationship between filial responsibilities and psychological wellbeing among Chinese adolescents raised in economically disadvantaged single-mother families. Furthermore, the Parental Acceptance-Rejection Theory (PARTheory; Rohner, 2004) posits that parental warmth is a beneficial parenting approach that fosters positive values and behaviors in adolescents (PARTheory; Rohner, 2004; Bacchini et al., 2023). Consequently, this study also assessed the moderating effects of maternal warmth on the relationship between filial responsibilities and adolescent psychological wellbeing.

## 2 Filial responsibilities and psychological wellbeing of adolescents

Within the realm of family studies and clinical practice, the parentification theory (Minuchin et al., 1967) is a prevailing theory that elucidates the relationship between filial responsibilities and psychological wellbeing in children and adolescents. The theory proposes that children assuming developmentally inappropriate parental roles within their family can lead to blurred parent-child boundaries and enmeshed relationships between the children and their parents (Boszormenyi-Nagy and Spark, 1973; Chase, 1999). Thus, it can be inferred that these “burdened children” (Chase, 1999) forfeit their childhood to fulfill their “parental” roles (Jurkovic, 1997). Empirical evidence suggests that parentification can result in adolescent distress and pathologies such as excessive guilt, anxiety, and somatic disorder (Chase, 1999; Koerner et al., 2004; McMahon and Luthar, 2007; Jankowski et al., 2011).

However, we contend that filial responsibilities do not necessarily impede adolescent wellbeing in Chinese society. The social identity theory (Hogg, 2003) adopts a socio-cultural perspective, viewing family responsibilities as normative processes for adolescent children to contribute to their family, which are essential for developing one’s family identity and fostering family solidarity (Fuligni and Zhang, 2004; Fuligni and Flook, 2005). This theory is particularly pertinent in explaining the significance of filial responsibilities in Chinese culture, where familism and collectivism are emphasized (Chao and Tseng, 2002). Filial piety is a central pillar for intergenerational conduct in the Chinese cultural system (Hwang, 1999). Children are expected to obey their parents’ instructions, provide tangible and emotional support to them, fulfill their wishes, bring honor to the family, and avoid tarnishing the family name (Ho, 1996). Under this framework, filial responsibilities are crucial for adolescents to establish their family identities and close affiliations with family members (Fuligni and Flook, 2005), which may enhance their positive psychological wellbeing when performing filial responsibilities (Leung and Shek, 2016). Previous studies in Chinese families have demonstrated that adolescents exhibit better positive psychological wellbeing when they are filial (Fuligni and Flook, 2005; Leung and Shek, 2016; Yang et al., 2024). Thus, it is essential to investigate the relationship of filial responsibilities and adolescent psychological wellbeing in Chinese single-mother families experiencing economic disadvantage.

### 2.1 Maternal warmth as a moderator

According to the Parental Acceptance-Rejection Theory (PARTheory; Rohner, 2004), parental warmth is a positive parenting process, whereby parents express their emotional bond to their children through physical, verbal, and symbolic behaviors (Rohner et al., 2012). It acts as a protective factor that enhances positive behavior and reduces negative developmental outcomes in adolescents (De Angelis et al., 2021). Additionally, the need-based theory of value acquisition (Kasser, 2002) may also explain the moderating role of maternal warmth in the relationship between filial responsibilities and adolescent psychological wellbeing. This theory suggests that maternal warmth may fulfill adolescents’ basic needs of relatedness and autonomy, thereby enhancing adolescents’ orientation to serve others (Bacchini et al., 2023), which may improve adolescents’ psychological wellbeing. Moreover, when adolescent children perceive high levels of maternal warmth, they may establish close mother-child affiliations and view filial responsibilities as gratitude toward their mother (Leung and Shek, 2016). The positive connotations of filial responsibilities would promote their psychological wellbeing. Conversely, when adolescent children perceive low levels of maternal warmth, they may view filial responsibilities as stress and strain (McMahon and Luthar, 2007) rather than gratitude toward their mother. Moreover, they may develop a sense of unfairness if they do not receive reciprocal response from their mother, which may lead to adolescent distress (Kuperminc et al., 2009). Theoretically, it is important to examine the moderating role of maternal warmth in the relationship between filial responsibilities and adolescent psychological wellbeing.

### 2.2 Do adolescent gender and age matter in the moderating effect of maternal warmth?

The socialization of family roles exhibits gender differences, particularly within Chinese families. Boys are typically socialized to become the family’s breadwinner, while girls are socialized to manage the household, as encapsulated by the common saying, “Nan zhu wai, nu zhu nei” (men work outside and women work inside the family) (Leung and Shek, 2021). Generally, adolescent girls undertake more instrumental and emotional filial responsibilities than boys (East, 2010) and have a stronger emotional attachment to their mothers (Updegraff et al., 2009). As such, girls may exhibit better wellbeing in performing more filial responsibilities than boys when they perceive higher levels of maternal warmth. However, in single-mother families, adolescent boys often assume “pseudo-father” roles, leading to increased anxiety levels (Leung et al., 2023). Maternal warmth is crucial for these boys to receive positive feedback, which validates their contributions. Therefore, it is essential to investigate whether the interaction effects between filial responsibilities and maternal warmth on adolescent wellbeing differ between boys and girls. Moreover, as girls typically invest more effort in family matters (Shek et al., 2022), parental warmth may not strongly motivate them to engage more in filial responsibilities. Thus, we anticipate that the relationship between filial responsibilities and wellbeing would be moderated in boys but not girls.

Additionally, filial responsibilities can become burdensome for adolescents who need to fulfill household chores and provide emotional support to their mother and siblings (McMahon and Luthar, 2007; Sy and Romero, 2008). Older adolescents may struggle more with these responsibilities than younger ones, as they need to devote more time to their studies and social life (Sy and Romero, 2008; East, 2010). However, older adolescents are often motivated by reciprocal filial piety (i.e., children’s repayment as gratitude for their parents’ effort) (Yeh and Bedford, 2003). Therefore, adolescents who perceive higher maternal warmth may be highly motivated to perform filial responsibilities to reciprocate their mother’s love and support (Cheah et al., 2012), potentially enhancing their psychological wellbeing. We expect that older adolescents performing more filial responsibilities will exhibit better psychological wellbeing when they perceive higher levels of maternal warmth, compared to younger adolescents.

### 2.3 The present study

The current study aimed to examine the associations of instrumental and emotional filial responsibilities with psychological wellbeing among Chinese adolescents from impoverished single-mother families, proposing maternal warmth as a moderator. The study posed four research questions:

Research question 1: What are the associations of instrumental and emotional filial responsibilities with psychological wellbeing (indexed by life satisfaction, anxiety and depression) among Chinese adolescents from impoverished single-mother families?

Hypotheses 1: Based on the social identity theory, we hypothesize that instrumental and emotional filial responsibilities will be associated with higher levels of life satisfaction (H1a and H1b), but lower levels of anxiety (H1c and H1d) and depression (H1e and H1f) among Chinese adolescents from impoverished single-mother families.

Research question 2: Does maternal warmth moderate the associations of instrumental and emotional filial responsibilities with wellbeing (indexed by life satisfaction, anxiety and depression) among Chinese adolescents from impoverished single-mother families?

Hypotheses 2: We hypothesize that, compared to adolescents perceiving lower levels of maternal warmth, the positive associations of instrumental and emotional filial responsibilities with life satisfaction will be stronger (H2a and H2b), and the negative associations with anxiety (H2c and H2d) and depression (H2e and H2f) will be weaker among Chinese adolescents perceiving higher levels of maternal warmth.

Research question 3: Does adolescent gender moderate the interactive effects between instrumental filial responsibilities and maternal warmth on wellbeing, and those between emotional filial responsibilities and maternal warmth on wellbeing, among Chinese adolescents from impoverished single-mother families?

Hypotheses 3: We hypothesize that, at higher levels of maternal warmth, boys performing higher levels of instrumental and emotional filial responsibilities will exhibit higher levels of life satisfaction (H3a and H3b), and lower levels of anxiety (H3c and H3d) and depression (H3e and H3f) than girls.

Research question 4: Does adolescent age moderate the interactive effects between instrumental filial responsibilities and maternal warmth on wellbeing, and those between emotional filial responsibilities and maternal warmth on wellbeing, among Chinese adolescents from impoverished single-mother families?

Hypotheses 4: We hypothesize that, at higher levels of maternal warmth, older adolescents performing higher levels of instrumental and emotional filial responsibilities will exhibit higher levels of life satisfaction (H4a and H4b), lower levels of anxiety (H4c and H4d) and depression (H4e and H4f) than younger adolescents.

This study is unique in several respects. Firstly, it focuses on adolescent children from impoverished Chinese single-mother families, a group often overlooked due to social stigma. Indeed, studies examining filial responsibilities in adolescents from impoverished Chinese single-mother families are scarce. Secondly, it is unclear whether the parentification theory (Minuchin et al., 1967) or role identity theory (Fuligni and Flook, 2005) better explains the relationship between adolescents’ filial responsibilities and their psychological wellbeing. Thirdly, this study delves deeper into the exploration of the moderating role of maternal warmth in the associations between filial responsibilities and adolescent wellbeing, thereby lending support to Rohner’s (2004) PARTheory. Fourthly, the study broadens its scope to scrutinize whether the child’s gender and age will moderate the moderating effects of maternal warmth in the associations between filial responsibilities and adolescent wellbeing.

## 3 Materials and methods

### 3.1 Participants

As a comprehensive list of Hong Kong single-mother families was unavailable, we recruited 325 adolescents from impoverished single-mother families through various social service centers in Hong Kong. We invited six non-governmental organisations (NGOs) that provide extensive social services in Hong Kong, including family service centers, school social work services, and children and youth service centers. We sent invitation letters to the directors of these NGOs, who accepted the invitations and assigned suitable service units to participate. Ultimately, 19 social service centers across Hong Kong, affiliated with these multi-service NGOs, participated in the study. The respondents were primarily members of these social service centers. The inclusion criteria for respondents were: (1) living in single-mother families; (2) aged between 11 and 17; and (3) a monthly household income at 50% of the median monthly domestic household income of Hong Kong (i.e., the official poverty threshold). Adolescents receiving Comprehensive Social Security Assistance (CSSA; a means-tested financial assistance offered by the Hong Kong Government to provide a subsistence of living for individuals and families facing economic hardship) were eligible for the study. However, adolescents receiving intensive counseling services were excluded from the research.

The sample comprised 141 girls (43.3%), with a mean age of 13.5 (SD = 2.10). Regarding educational level, 67 (21.3%) were primary school students, 182 (56.0%) were junior secondary school students, and 65 (20.0%) were senior secondary school students (11, 3.4% did not respond). Of the respondents, 123 (37.8%) were the only children in their family, and 86 (26.5%) were the eldest children. In terms of family background, 222 (68.3%) respondents came from divorced families, 23 (7.1%) from separated families, 45 (13.8%) from widowed families, and 29 (7.1%) reported other circumstances (e.g., lost contact, etc.) (6 did not respond). Most respondents belonged to impoverished families, with 209 (64.3%) being CSSA recipients. Regarding maternal educational levels, the majority were junior secondary levels or lower (*n* = 183, 56.7%). A total of 113 mothers had a senior secondary level education (*n* = 113, 34.8%), and only a few (*n* = 27, 8.3%) had post-secondary level education (*n* = 2, 1.9% did not respond). For maternal occupation, a high proportion (*n* = 212, 65.2%) were house-parents, 52 (16.5%) mothers were engaged in low-skilled jobs, and 14 (4.3%) were unemployed. Only seven (2.1%) mothers were engaged in managerial and professional jobs.

### 3.2 Procedures

Social workers or research assistants, who had undergone appropriate training, approached adolescents and their mothers to explain the study’s purpose and their rights to participate voluntarily or withdraw from the study. Written informed consent was obtained from both parties. The respondents were given the option to complete the questionnaire at home or in social service centers. Adolescents completed an Adolescent Questionnaire, which included measurements of instrumental and emotional filial responsibilities, perceived maternal warmth, wellbeing attributes (indexed by life satisfaction, anxiety, and depression), and various demographic features (gender, age, educational level, family structure, sibling order, CSSA recipients, maternal mental health status, etc.) in a self-administered format. As a token of appreciation for their time and effort, respondents received a supermarket coupon worth HK$50 (US$6.4). The study received ethical approval from the Human Subjects Ethics Sub-committee of an internationally recognized university.

### 3.3 Measurements

Filial responsibilities: Filial Responsibilities Scale for Youth (FRS-Y; Kuperminc et al., 2009). Based on the FRS-Y developed by Kuperminc et al. (2009), we translated into Chinese and the measurements showed good psychometric properties in a sample of Chinese adolescents in Hong Kong (Leung et al., 2023). In this study, both Instrumental Filial Responsibilities Subscale (IFRS) and Emotional Filial Responsibilities Subscale (EFRS) were used to measure instrumental and emotional filial responsibilities, respectively. The respondents rated each item on a 4-point Likert scale from “1 = Strongly disagree” to “4 = Strongly agree.” The sample items of IFRS and EFRS are “I often do the laundry in my family,” and “If someone in my family is upset, I try to help in some way,” respectively. Higher mean scores of IFRS and EFRS indicate higher instrumental and emotional filial responsibilities, respectively. Both IFRS and EFRS showed good reliabilities in this study (IFRS: α = 0.81; EFRS: α = 0.87).

Maternal warmth: Short-form Egna Minnen av Barndoms Uppfostran (s-EMBU; Winefield et al., 1989). Based on the original EMBU that measures various child-rearing patterns, Winefield et al. (1989) developed a 27-item s-EMBU, which specifically assesses parental warmth, over-protection and rejection. A Chinese version of s-EMBU was translated and validated by Jiang et al. (2010) in the Chinese context, with good internal consistency and factorial validity. The Warmth Subscale of s-EMBU was used in the current study. A sample item reads “If things go badly for me, my mother tries to comfort and encourage me.” The respondents will rate the measurement in a four-point Likert scale, ranging from 1 = “Strongly disagree” to 4 = “Strongly agree.” Higher mean scores indicate higher levels of perceived maternal warmth. The Warmth Subscale of s-EMBU showed good internal consistency in this study (α = 0.91).

#### 3.3.1 Adolescent wellbeing

Life satisfaction: Satisfaction with Life Scale (SWLS). Based on original Satisfaction with Life Scale (SWLS; Diener et al., 1985), Shek (1992) translated it to measure one’s subjective appraisal of quality of life and showed good psychometric properties in Chinese adolescent samples in previous studies (e.g., Leung and Shek, 2020). A sample item is “In most ways my life is close to my ideal.” Each item was rated on a 6-point Likert scale from “1 = strongly disagree” to “6 = strongly agree.” Higher mean score of SWLS indicates higher life satisfaction (α = 0.86).

Anxiety and depression: Chinese Hospital Anxiety and Depression Scale (HADS-C). Based on the original Hospital Anxiety and Depression Scale (HADS) developed by Zigmond and Snaith (1983), Leung et al. (1993) translated it into Chinese version (HADS-C) and demonstrated acceptable psychometric properties in a Chinese sample (Leung et al., 1999). There are two subscales, including the Anxiety Subscale (7 items; sample item: “I feel tense or ‘wound up”’) and the Depression Subscale (7 items, sample item: “I have lost interest in my appearance”). The respondents will rate each item in a 4-point Likert scale from “0 = not at all” to “3 = most of the time.” Higher mean scores of Anxiety and Depression Subscales indicate higher levels of anxiety and depression, respectively. Both subscales demonstrated acceptable internal consistencies in the current study (Anxiety Subscale α = 0.77; Depression Subscale α = 0.67).

### 3.4 Data analysis

We first performed correlational analyses to assess the bivariate relationships among instrumental and emotional filial responsibilities, adolescent wellbeing (indexed by life satisfaction, anxiety and depression), and some psychosocial and demographic attributes of mothers (age, educational level, duration of stay in Hong Kong, mental health status), adolescents (e.g., gender, age and sibling order) and the family (number of children).

Hierarchical multiple regression analyses were performed to assess the relationship between instrumental filial responsibilities and life satisfaction, and the moderating effect of maternal warmth. Previous studies showed that age, educational level, duration of stay in Hong Kong and mental health status of mothers, and gender, age and sibling order of adolescents, and number of children in the family were related to filial responsibilities and/or their wellbeing (Jurkovic et al., 2005; McMahon and Luthar, 2007; Kuperminc et al., 2009; East, 2010), they were considered as the covariates. First, we entered covariates into the hierarchical regression equation. Next, we added instrumental filial responsibilities (i.e., the predictor) into the equation. Then, we put maternal warmth (i.e., the moderator) into the regression block. Finally, instrumental filial responsibilities and maternal warmth were mean-centered. We computed an interaction term of “instrumental filial responsibilities X maternal warmth” and input into the equation. The moderating effect was supported when the interaction term was significantly associated with adolescent life satisfaction. We further performed simple slope analyses (Cohen et al., 2003) to assess the relationship between instrumental filial responsibilities and adolescent life satisfaction at high levels (1 SD higher than the mean) and low levels (1 SD lower than the mean) of perceived maternal warmth.

Then, we examined whether boys and girls were different in the moderating effect of maternal warmth in the association of instrumental filial responsibilities with adolescent life satisfaction. Three interaction terms, “instrumental filial responsibilities × adolescent gender,” “maternal warmth × adolescent gender,” “instrumental filial responsibilities × maternal warmth × adolescent gender” were computed and added into the equation. If the regression of the interaction term of “instrumental filial responsibilities × maternal warmth × adolescent gender” was significantly related to adolescent life satisfaction, gender differences in the moderating effect of maternal warmth in the association of instrumental filial responsibilities with adolescent life satisfaction would be established.

We further performed identical procedure to test whether the moderating effect of maternal warmth in the relationship between instrumental filial responsibilities and life satisfaction were different between younger and older adolescents, using median spilt. Similar procedures were repeated to assess the moderating effects of maternal warmth in the linkage of instrumental filial responsibilities with other adolescent wellbeing outcomes (i.e., anxiety and depression), and those in the associations of emotional filial responsibilities with adolescent life satisfaction, anxiety and depression, respectively.

## 4 Results

Correlational analysis revealed a positive relationship between emotional filial responsibilities and adolescent life satisfaction, and a negative correlation with adolescent depression. However, instrumental filial responsibilities showed no significant correlation with adolescent life satisfaction, anxiety, or depression. Furthermore, maternal warmth was found to be associated with higher life satisfaction and lower anxiety and depression among adolescents. Additionally, adolescent girls, older adolescents, those with more siblings, and those with higher sibling order assumed more instrumental filial responsibilities, while older adolescents and those with lower sibling order assumed more emotional filial responsibilities (Table 1).

**TABLE 1:** Correlations of the measuring variables.

	Mean	SD	1	2	3	4	5	6	7	8	9	10	11	12	13
1. Instrumental filial responsibilities	1.89	0.78	1.00												
2. Emotional filial responsibilities	2.30	0.61	0.37***	1.00											
3. Perceived maternal warmth	2.92	0.63	0.19***	0.46***	1.00										
4. Life satisfaction	3.71	1.10	0.21	0.29***	0.53***	1.00									
5. Anxiety	2.09	0.06	0.00	-0.01	-0.29***	-0.46***	1.00								
6. Depression	1.97	0.54	-0.07	-0.19***	-0.52***	-0.50***	0.48***	1.00							
7. Adolescent gender (boys = 1; girls = 2)	1.43	0.50	0.13*	0.06	0.06	-0.05	0.11	0.02	1.00						
8. Adolescent age	13.53	2.10	0.13*	0.13*	0.01	-0.12*	0.08	0.02	-0.06	1.00					
9. No. of children in the family	1.76	0.77	0.44***	-0.05	-0.10	0.00	0.11	0.04	0.10	0.15**	1.00				
10. Sibling order	1.05	1.01	0.34***	-0.12*	-0.10	-0.00	0.12*	0.05	-0.06	0.03	0.72***	1.00			
11. Mother’s age	44.05	5.80	-0.05	0.05	0.09	-0.05	0.09	-0.16**	-0.07	0.28***	0.01	0.23***	1.00		
12. Mother’s educational level	3.38	0.90	-0.0.05	0.04	0.14*	-0.08	-0.00	-0.01	0.03	-0.03	-0.06	-0.09	-0.12*	1.00	
13. Mother’s duration of stay in Hong Kong	3.79	1.49	-0.0.05	-0.03	-0.05	-0.03	0.01	0.01	-0.02	0.11	0.00	0.03	0.10	-0.16**	1.00
14. Maternal distress	2.54	0.89	0.06	-0.00	-0.14*	-0.07	-0.12*	0.13*	0.03	-0.02	0.06	0.01	-0.08	-0.05	-0.11*

*p < 0.05,

**p < 0.01,

***p < 0.001.

**FIGURE 1:**
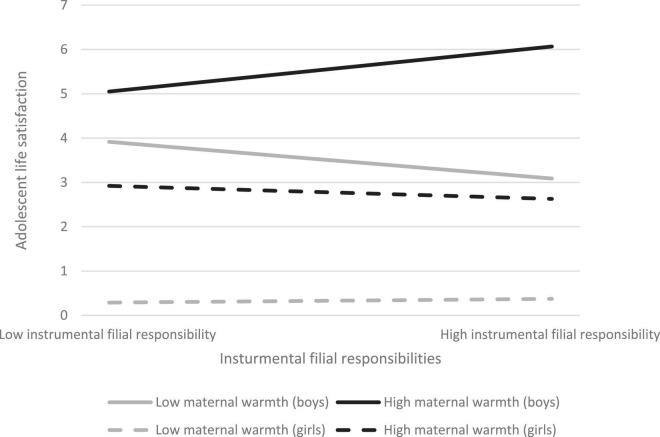
Regression of adolescent life satisfaction by instrumental filial responsibilities in high and low levels of parental warmth between adolescent boys and girls.

Regarding adolescent life satisfaction as an outcome, the prediction of instrumental filial responsibilities on adolescent life satisfaction was significant after controlling the covariates, with β = 0.24 (*p* < 0.001), supporting H1a. The interaction term of “instrumental filial responsibilities X perceived maternal warmth” was positively associated with adolescent life satisfaction (β = 0.10, *p* < 0.05), indicating that perceived maternal warmth acted as a moderator (Table 2), supporting H2a. Further analysis showed a significant negative association of the interaction term of “instrumental filial responsibilities X perceived maternal warmth X gender” with adolescent life satisfaction (β = −0.15, *p* < 0.01) (Table 2), indicating a difference between adolescent boys and girls in the moderation of maternal warmth in the association of instrumental filial responsibilities with adolescent life satisfaction. In general, adolescent boys exhibited higher life satisfaction when they performed more instrumental filial responsibilities than did girls. However, adolescent boys who perceived higher levels of maternal warmth exhibited higher life satisfaction when they performed more instrumental filial responsibilities (β = 0.33, *p* < 0.01) (Table 3). Conversely, boys who perceived lower levels of maternal warmth expressed lower life satisfaction when they performed more instrumental filial responsibilities (β = −0.32, *p* < 0.05) (Table 3). However, the association of instrumental filial responsibilities with life satisfaction among adolescent girls was non-significant regardless of degree of maternal warmth they perceived (Figure 1). H3a was supported. Regression analyses showed the moderating effect of maternal warmth in the relationship between instrumental filial responsibilities and adolescent life satisfaction did not differ between younger and older adolescents (Table 2). H4a was not supported.

**TABLE 2:** Regression of adolescent psychological wellbeing by instrumental filial responsibilities in the context of maternal warmth.

	Life satisfaction			Anxiety			Depression		
	*b*	SE	β	*B*	SE	β	*b*	SE	β
**Step 1**									
Gender of adolescents	-0.18	0.14	-0.08	0.18	0.07	0.17**	0.08	0.07	0.08
Age of adolescents	-0.08	0.04	-0.15*	0.02	0.02	0.09	0.03	0.02	0.11
Sibling order	-0.06	0.10	-0.05	0.06	0.05	0.11	0.09	0.05	0.17
No. of children	0.11	0.13	0.08	0.02	0.07	0.02	-0.08	0.06	-0.12
Mother’s education	-0.09	0.08	-0.07	0.02	0.04	0.04	0.00	0.04	0.00
Mother’s age	-0.01	0.01	-0.05	0.00	0.01	0.04	-0.02	0.01	-0.23
Mother’s migration status	-0.04	0.05	-0.05	0.05	0.02	0.14*	0.04	0.02	0.12
Maternal distress	-0.08	0.08	-0.07	0.05	0.04	0.08	0.05	0.04	0.09
**Step 2**									
Instrumental filial responsibilities	0.34	0.10	0.24***	-0.07	0.05	-0.10	-0.15	0.05	-0.22***
**Step 3**									
Perceived maternal warmth	1.03	0.09	0.61***	-0.27	0.05	-0.32***	-0.42	0.05	-0.51***
**Step 4**									
Instrumental filial responsibilities X perceived maternal warmth	0.21	0.11	0.10*	-0.07	0.06	-0.07	0.03	0.05	0.03
**Gender as a moderator**									
**Step 5a**									
Instrumental filial responsibilities X gender	-0.08	0.15	-0.03	0.03	0.09	0.02	0.10	0.08	0.07
Perceived maternal warmth X gender	0.29	0.18	0.09	-0.17	0.10	-0.10	-0.01	0.09	-0.01
**Step 6a**									
Instrumental filial responsibilities X perceived maternal warmth X gender	-0.58	0.22	-0.15**	-0.17	0.13	-0.09	-0.12	0.11	-0.07
**Age as a moderator**									
**Step 5b**									
Instrumental filial responsibilities X age	0.00	0.04	-0.01	0.03	0.02	0.09	-0.02	0.02	-0.05
Perceived maternal warmth X age	0.04	0.04	0.05	-0.01	0.02	-0.03	-0.01	0.02	-0.03
**Step 6b**									
Instrumental filial responsibilities X perceived maternal warmth X age	-0.01	0.05	-0.01	-0.02	0.03	-0.05	0.00	0.03	0.00

*p < 0.05,

**p < 0.01,

***p < 0.001.

**TABLE 3:** Simple slope analyses of the prediction of filial responsibilities on adolescent psychological wellbeing with adolescent gender, age and maternal warmth as moderators.

			Regression coefficient (β)		
Moderator		Predictor	Overall	Boys	Girls
			Life satisfaction		
Maternal warmth	Higher level (+1 SD)	Instrumental filial responsibilities	0.12	0.33**	-0.11
	Lower level (−1 SD)		-0.16	-0.32*	0.03
Maternal warmth	Higher level (+1 SD)	Emotional filial responsibilities	0.22*	N.A.	N.A.
	Lower level (−1 SD)		-0.04	N.A.	N.A.
			**Overall**	**Older adolescents**	**Younger adolescents**
			**Anxiety**		
Maternal warmth	Higher level (+1 SD)	Emotional filial responsibilities	-0.25*	-0.36**	-0.05
	Lower level (−1 SD)		0.33**	0.47***	-0.14

*p < 0.05,

**p < 0.01,

***p < 0.001.

**FIGURE 2:**
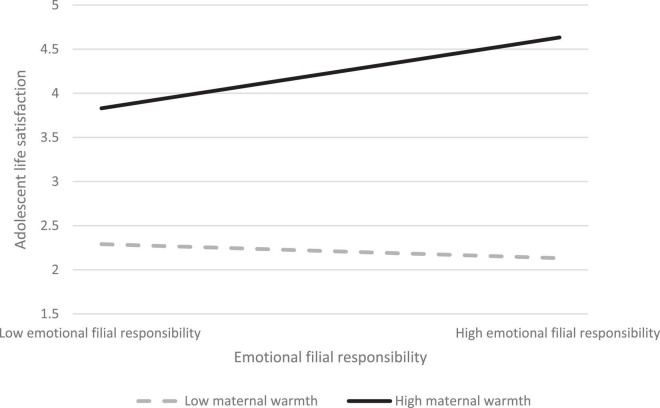
Regression of adolescent life satisfaction by emotional filial responsibilities in high and low levels of maternal warmth.

Moreover, the regression of emotional filial responsibilities on adolescent life satisfaction was significant (β = 0.38, *p* < 0.001), supporting H1b. The interaction term of “emotional filial responsibilities X perceived maternal warmth” was positively associated with adolescent life satisfaction (β = 0.11, *p* < 0.05) (Table 2), indicating that perceived maternal warmth also moderated the association of emotional filial responsibilities with adolescent life satisfaction (Table 4). H2b was supported. Adolescents who perceived higher maternal warmth reported greater life satisfaction when they performed more emotional filial responsibilities (β = 0.22, *p* < 0.05) (Table 3). Conversely, the relationship between emotional filial responsibilities and adolescent life satisfaction was non-significant (β = −0.04, *p* > 0.05) (Table 3) when they perceived lower levels of maternal warmth (Figure 2). Regression analyses showed that there was no effect of adolescent gender and age on the moderating effect of maternal warmth in the association of emotional filial responsibilities and adolescent life satisfaction (Table 4). H3b and H4b were not supported.

**TABLE 4:** Regression of adolescent psychological wellbeing by emotional filial responsibilities in the context of maternal warmth.

	Life satisfaction			Anxiety			Depression		
	*b*	SE	β	*B*	SE	β	*b*	SE	β
**Step 1**									
Gender of adolescents	-0.20	0.14	-0.09	0.18	0.07	0.16**	0.08	0.07	0.08
Age of adolescents	-0.09	0.04	-0.17	0.02	0.02	0.08	0.03	0.02	0.13
Sibling order	-0.07	0.10	-0.06	0.05	0.05	0.09	0.09	0.05	0.17
No. of children	0.13	0.13	0.09	0.02	0.07	0.03	-0.09	0.06	-0.13
Mother’s education	-0.08	0.08	-0.07	0.03	0.04	0.05	0.00	0.04	0.01
Mother’s age	-0.01	0.01	-0.03	0.01	0.01	0.07	-0.02	0.01	-0.24***
Mother’s migration status	-0.04	0.05	-0.05	0.06	0.02	0.16*	0.05	0.02	0.13*
Maternal distress	-0.07	0.08	-0.05	0.06	0.04	0.10	0.05	0.04	0.09
**Step 2**									
Emotional filial responsibilities	0.69	0.11	0.38***	-0.12	0.06	-0.13*	-0.24	0.05	-0.27***
**Step 3**									
Perceived maternal warmth	0.96	0.10	0.58***	-0.28	0.06	-0.34***	-0.42	0.05	-0.52***
**Step 4**									
Emotional filial responsibilities X Perceived maternal warmth	0.25	0.12	0.11*	-0.27	0.07	-0.24***	-0.04	0.06	-0.04
**Gender as a moderator**									
**Step 5a**									
Emotional filial responsibilities X gender	0.09	0.21	0.02	0.03	0.12	0.01	-0.08	0.11	-0.05
Perceived maternal warmth X gender	0.25	0.19	0.07	-0.13	0.11	-0.08	0.07	0.10	0.05
**Step 6a**									
Emotional filial responsibilities X perceived maternal warmth X gender	-0.08	0.25	-0.02	-0.11	0.14	-0.05	-0.21	0.13	-0.10
**Age as a moderator**									
**Step 5b**									
Emotional filial responsibilities X age	0.05	0.05	0.05	0.02	0.03	0.04	-0.06	0.03	-0.14*
Perceived maternal warmth X age	0.02	0.05	0.02	0.00	0.03	0.01	0.02	0.03	0.06
**Step 6b**									
Emotional filial responsibilities X perceived maternal warmth X age	0.02	0.06	0.02	-0.07	0.04	-0.15*	0.01	0.03	0.02

*p < 0.05,

**p < 0.01,

***p < 0.001.

**FIGURE 3:**
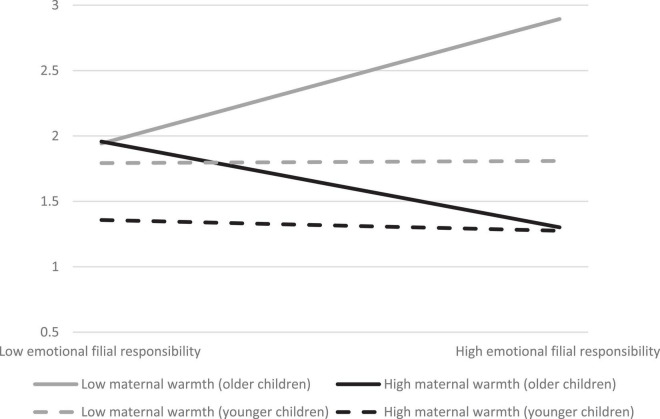
Regression of adolescent anxiety by emotional filial responsibilities in high and low levels of parental warmth between younger and older adolescents.

For adolescent anxiety, the prediction of instrumental filial responsibilities on adolescent anxiety was not significant (β = −0.10, *p* > 0.05) (Table 2). H1c was not supported. Maternal warmth did not moderate the association of instrumental filial responsibilities and adolescent anxiety (Table 2). H2c was not supported. However, the prediction of emotional filial responsibilities on adolescent anxiety was significant (β = −0.13, *p* < 0.05). H1d was supported. The interaction term of “emotional filial responsibilities X perceived maternal warmth” was negatively associated with adolescent anxiety (β = −0.24, *p* < 0.001), suggesting that perceived maternal warmth was a moderator altering the relationship between emotional filial responsibilities and adolescent anxiety. H2d was supported. Further analyses showed that the interaction term of “emotional filial responsibilities X perceived maternal warmth X age” was significantly associated with adolescent anxiety (β = −0.15, *p* < 0.05) (Table 4), indicating a difference between younger and older adolescents in the moderating effect of maternal warmth in the association of emotional filial responsibilities and adolescent anxiety. H4d was supported. When older adolescents perceived higher levels of maternal warmth, they reported lower levels of anxiety when they performed more emotional filial responsibilities (β = −0.36, *p* < 0.01) (Table 3). On the contrary, those who perceived lower levels of maternal warmth exhibited higher anxiety when they performed more emotional filial responsibilities (β = 0.47, *p* < 0.001). However, the association of emotional filial responsibilities with anxiety was non-significant in younger adolescents, regardless of their perceptions on maternal warmth (Figure 3). Moreover, the moderating effect of maternal warmth in the association of emotional filial responsibilities with anxiety did not differ between adolescent boys and girls (Table 4). H3d was not supported.

Regarding adolescent depression as an outcome, the prediction of instrumental and emotional filial responsibilities on adolescent depression was significant, with β = −0.22 (*p* < 0.001) (Table 2) and −0.27 (*p* < 0.001) (Table 4), respectively. H1e and H1f were supported. However, perceived maternal warmth did not moderate the associations of both instrumental and emotional filial responsibilities with adolescent depressive symptoms, respectively (Tables 2, 4). H2e and H2f were not supported.

## 5 Discussion

The present study examined the moderating effect of maternal warmth in the associations of filial responsibilities with adolescent psychological wellbeing (indexed by life satisfaction, anxiety, and depression) among Chinese adolescents raised in poor single-mother families. The literature based on parentification theory (Minuchin et al., 1967; Chase, 1999) and role identity theory (Fuligni and Flook, 2005) provides diverse views on whether filial responsibilities enhance or hamper adolescent psychological wellbeing. The results showed some support for role identity theory that instrumental and emotional filial responsibilities were positively associated with adolescent life satisfaction and negatively linked to adolescent depression. Emotional filial responsibilities were also negatively associated with adolescent anxiety. Chinese adolescents may consider instrumental and emotional filial responsibilities as normative family responsibilities that each family member needs to fulfill (Fuligni and Zhang, 2004), particularly when their families are facing poverty and single parenthood. Moreover, filial responsibilities indicate their worthiness in providing support to the family, which is important for enhancing their family identity and building family cohesion (Fuligni and Flook, 2005).

However, family context and adolescents’ demographic characteristics matter on whether adolescents appreciate or are burdened by performing filial responsibilities. Maternal warmth is a necessary condition for adolescents to build up their willingness to serve the family, which echoes Acceptance-Rejection Theory (Rohner, 2004) as well as previous studies drawing on this framework (i.e., De Angelis et al., 2021; Bacchini et al., 2023). When adolescents perceive higher maternal warmth, they develop a sense of gratitude toward their mother, and are more filial to repay their mother (Leung and Shek, 2016). Besides, adolescents may feel that their contributions are recognized by their mother (Elliott, 2009), which is important for their mental health (Vélez et al., 2020). Hence, maternal warmth contributes to the meaning-making process of adolescent children in evaluating their worthiness of performing filial duties (Bacchini et al., 2023). Conversely, when adolescents perceive lower maternal warmth, they may perceive filial responsibilities as family burdens that they are forced to do, which may create a sense of unfairness and blame for their mother (Jurkovic et al., 2005). Besides, the feelings of unworthiness and feelings of hurt from parental rejection may certainly reduce the positive association of filial responsibilities on adolescent psychological wellbeing (Rohner et al., 2012; Yang et al., 2024).

It is important to note that the child’s gender moderated the effect of maternal warmth on the relationship between instrumental filial responsibilities and adolescent life satisfaction. Maternal warmth amplified the correlation between instrumental filial responsibilities and life satisfaction in boys, but not in girls. In single-mother families, sons often assume the role of a “surrogate father,” taking care of siblings and managing family affairs (Weiss, 1979; McLanahan and Sandefur, 2009). Boys’ perception of heightened maternal warmth may be interpreted as maternal validation of their familial roles and contributions, potentially enhancing their life satisfaction. Conversely, lower perceived maternal warmth may be seen as maternal negligence of their roles and contributions, potentially diminishing their life satisfaction. Girls, in comparison to boys, are typically socialized to undertake more instrumental filial responsibilities at home and are more adaptable to this role (East, 2010). Therefore, they may adhere more to this gender stereotype and assume these family roles, regardless of their perceptions of maternal warmth. Furthermore, it is important to note that child gender did not moderate the effects of maternal warmth on the relationship between emotional filial responsibilities and adolescent psychological wellbeing. Providing emotional support to family members and alleviating negative emotions are challenging tasks for adolescents, irrespective of their gender. Maternal warmth is indeed crucial for adolescents to foster cohesion and find meaning in their contributions, for both boys and girls.

The results also indicated that older adolescents who perceived higher maternal warmth experienced less anxiety when they performed more emotional filial responsibilities. Conversely, those who perceived lower maternal warmth reported more anxiety when they performed more emotional filial responsibilities. However, the relationship between emotional filial responsibilities and anxiety among younger adolescents remained stable, regardless of their perceptions of maternal warmth. A possible explanation is that older adolescents are primarily driven by reciprocal filial piety (i.e., children’s repayment as gratitude for their parents’ effort), whereas younger adolescents are driven by authoritative filial piety (i.e., children’s obedience due to social convention) (Yeh and Bedford, 2003). Therefore, older adolescents may be more sensitive to their mother’s recognition of their contributions and may expect a positive reciprocal response from their mother when they perform emotional filial responsibilities. However, a lack of maternal warmth may be perceived by adolescents as maternal invalidation of their contributions, potentially leading to stress, self-doubt, and anxiety (Yap et al., 2008). Conversely, younger adolescents may have a simpler motive to perform emotional filial responsibilities out of authoritarian filial piety. As studies on filial responsibilities, filial piety, and adolescent anxiety are limited at different ages of children in Chinese families, further research in this area is necessary.

## 6 Theoretical and practical implications

Previous studies have consistently shown that adolescents growing up in impoverished single-parent families are susceptible to mental health problems (Amato, 2005; Langton and Berger, 2011; Chapple, 2013). However, few studies have examined the contribution of adolescent children to their families in adapting to structural and economic changes, and their impacts on adolescent psychological wellbeing. The current study broadens the scope of previous studies by examining the associations of filial responsibilities with psychological wellbeing among Chinese adolescents living in impoverished single-mother families, as well as the moderating role of maternal warmth in these associations. The study provides some support for the social identity theory, suggesting that Chinese adolescents exhibited higher life satisfaction and fewer depressive symptoms when performing filial responsibilities. Furthermore, the findings also resonate with the Parental Acceptance-Rejection Theory (Rohner, 2004), identifying that maternal warmth served as a moderator that moderated the relationships between instrumental and emotional filial responsibilities and adolescent life satisfaction, respectively, and between emotional filial responsibilities and adolescent anxiety. Moreover, adolescent gender moderated the effect of maternal warmth on the relationship between instrumental filial responsibilities and adolescent life satisfaction, and adolescent age moderated the effect of maternal warmth on the relationship between emotional filial responsibilities and adolescent anxiety. The findings are novel in identifying the family and demographic factors that affect the positive associations of filial responsibilities and psychological wellbeing among Chinese adolescents living in impoverished single-mother families. Theoretically, it facilitates the development of Chinese family models for these underprivileged families that are under-researched in the family literature.

Practically, the study provides insights for social workers, family practitioners, and youth workers on how to assist adolescents and their families in overcoming the disruptions caused by poverty and single motherhood. The study showed that filial responsibilities were protective factors that promoted adolescent psychological wellbeing. However, promoting maternal warmth is indeed a necessary condition for adolescent children to find meaning in their filial responsibilities, which may affect their psychological wellbeing. Moreover, it is important to pay more attention to adolescent boys who perform instrumental filial responsibilities, as well as older adolescents who perform emotional filial responsibilities for their family. The “surrogate parent” roles may bring pressure to adolescents (McLanahan and Sandefur, 2009), particularly those perceiving lower levels of maternal warmth. Family interventions such as family resilience programs are necessary to enable mothers to recognize their children’s contributions to the family and exercise maternal warmth for their children. Additionally, policymakers may need to pay more attention to the needs of impoverished single-mother families in Hong Kong. Adolescents may need to contribute more to maintain proper family functioning in single-parent families. Single mothers may also face numerous challenges in recovering from divorce or widowhood, and poverty. Tangible (e.g., family aide service) and non-tangible (counseling services) resources are needed to assist these families in adapting to the changes.

## 7 Limitations of the study

This study is subject to several limitations. Firstly, its cross-sectional nature restricts the ability to infer causality between filial responsibilities, maternal warmth, and adolescent psychological wellbeing, necessitating future longitudinal studies. Secondly, the sample was sourced from social service centers in Hong Kong due to the unavailability of a comprehensive list of single-parent families and the difficulties in identifying adolescents from impoverished single-mother families. The pandemic-induced school closures further complicated data collection. This sampling strategy, however, excluded adolescents not receiving community social services, potentially a more vulnerable group lacking social support. Future studies should consider recruiting these specific samples from schools. Thirdly, the study’s sample comprised Chinese adolescents from impoverished single-mother families. While adolescents are the recipients of maternal warmth (Elstad and Stefansen, 2014), and their subjective perception of this warmth is crucial for their psychological wellbeing, it would be methodologically advantageous to include mothers’ reports for a more objective and comprehensive assessment. Fourthly, the study was conducted during the COVID-19 pandemic, a period that may have affected family dynamics (Shek et al., 2023) and introduced additional stressors impacting adolescent psychological wellbeing. Replication of the study post-pandemic is recommended. Finally, as the study was conducted in Hong Kong, similar studies should be conducted in other Chinese communities (e.g., Mainland China, American Chinese, etc.) and cross-cultural studies with non-Chinese communities (e.g., the US, other Asian countries) should be considered.

## 8 Conclusion

While most studies on single-mother families primarily focus on parents’ efforts to adjust to changes in family structure (Murry et al., 2001; Daryanani et al., 2016; Leung and Shek, 2018), this study acknowledges the significant contributions of adolescent children in adapting to these changes. It pioneers the examination of the relationship between filial responsibilities and psychological wellbeing among Chinese adolescents from impoverished single-mother families. The findings reveal a positive correlation between instrumental and emotional filial responsibilities and adolescent life satisfaction, and a negative correlation with adolescent depression. However, maternal warmth moderated the relationship between instrumental filial responsibilities and adolescent life satisfaction among boys, and between emotional filial responsibilities and adolescent life satisfaction. Maternal warmth also significantly altered the negative relationship between emotional filial responsibilities and adolescent anxiety in older adolescents. These findings offer valuable insights for developing theoretical models related to single-motherhood and poverty in Chinese families, and for formulating suitable social services to assist adolescents from impoverished single-mother families. As McMahon and Luthar (2007) noted, “the critical problem may not be the extent to which children are involved in caregiving but the extent to which the caregiving occurs in an unsupportive, unresponsive, invalidating family environment” (p. 280). This study supports their assertion by highlighting the moderating role of maternal warmth in the relationship between filial responsibilities and psychological wellbeing among Chinese adolescents from impoverished single-mother families.

## Data availability statement

The raw data supporting the conclusions of this article will be made available by the authors, without undue reservation.

## Ethics statement

The studies involving humans were approved by the Human Subjects Ethics Sub-committee (HSESC) (or its Delegate) of The Hong Kong Polytechnic University. The studies were conducted in accordance with the local legislation and institutional requirements. Written informed consent for participation in this study was provided by the participants’ legal guardians/next of kin.

## Author contributions

JL: Conceptualization, Formal analysis, Funding acquisition, Methodology, Project administration, Writing – original draft. DS: Formal analysis, Funding acquisition, Methodology, Writing – review & editing.
